# Supplementary material for: Climate-based modelling and forecasting of dengue in three endemic departments of Peru
Source: PLoS Negl Trop Dis. 2024 Dec 4;18(12):e0012596. doi: 10.1371/journal.pntd.0012596 (PMC11649132; doi:10.1371/journal.pntd.0012596)
Supplement: S1 Appendix — Table A: Summary of acronyms used throughout the analysis. Table B: Summary statistics for the studied Peruvian departments. Population refers to the last mid-year population estimate of a department’s population within the studied period, as of 30 June 2021, whilst Population Density is the corresponding estimated population per km2. DIR is the mean dengue incidence rate per 100,000 across the studied period of 2010 to 2021. Maximum Temperature and Minimum Temperature similarly relate to the mean average observed value of the monthly means of maximum daily temperatures and of the monthly means of minimum daily temperatures. Total Precipitation is the mean of monthly observed total precipitations. Table C: Summary statistics for LOOCV outbreak detection performance. Out-of-sample observations (n = 417), across 2010 to 2021, were classified as being predicted true outbreaks such that DIR exceeded 50 (or 150) per 100,000 if the posterior probability of DIR exceeding 50 (or 150) per 100,000 was greater than a calibrated cut-off probability of 0.16 (or 0.13). True Positive measures the hit rate, or equivalently the proportion of true outbreaks correctly detected, whilst False Positive measures the proportion of non-outbreaks incorrectly classified as outbreaks. Accuracy represents the proportion of observations whose outbreak classification matched the true observed state (such as a predicted outbreak coinciding with an outbreak). Precision measures the proportion of predicted outbreaks which were true outbreaks. Finally, the AUC is the area under the Receiver Operating Characteristic (ROC) curve, is used as a measure of our model’s skill for distinguishing between outbreaks and non-outbreaks, and here, represents an upper bound on the model’s retrospective performance (due to calibration of our cut-off probability for outbreak classification). 95% confidence intervals (CIs) for summary statistics were derived using exact binomial confidence limits, with the exception o [file pntd.0012596.s001.pdf]

S1 Appendix

Section 1 Acronyms

Table A: **Summary of acronyms used throughout the analysis.**

**Abbreviation**	Definition
Ae. aegypti	*Aedes aegypti*
**ACF**	Autocorrelation Function
**AUC**	Area Under the Curve
**BYM**	Besag, York and Mollié
**CV**	Cross-Validated
**DIC**	Deviance Information Criterion
**DIR**	Dengue Incidence Rate
**DLNM**	Distributed Lag Non-linear Models
**ENSO**	El Niño Southern Oscillation
**ICEN**	El Niño Coastal Index
**IGP**	Geophysical Institute of Peru
**INEI**	National Institute of Statistics and Information of Peru
**INLA**	Integrated Nested Laplace Approximation
**LOOCV**	Leave-One-Out-Cross-Validation
**MAE**	Mean Absolute Error
**ONI**	Oceanic Niño Index
**PIT**	Probability Integral Transform
**prec**	Precipitation
**ROC**	Receiver Operating Characteristic
**RR**	Relative Risk
**RSI**	Relative Strength Index
**RW**	Random Walk
**SPI-6**	Standardized Precipitation Index (6-Month)
**SST**	Sea Surface Temperature
**tmax**	Maximum Temperature
**tmin**	Minimum Temperature

Section 2   Further study area details

Table B: **Summary statistics for the studied Peruvian departments.** *Population* refers to the last mid-year population estimate of a department's population within the studied period, as of 30 June 2021, whilst *Population Density* is the corresponding estimated population per km^2. *DIR* is the mean dengue incidence rate per 100,000 across the studied period of 2010 to 2021. *Maximum Temperature* and *Minimum Temperature* similarly relate to the mean average observed value of the monthly means of maximum daily temperatures and of the monthly means of minimum daily temperatures. *Total Precipitation* is the mean of monthly observed total precipitations.

**Department**	Population	Population Density	DIR	Maximum Temperature	Minimum Temperature	Total Precipitation
**Lambayeque**	1,325,912	93·0	4·1	27·6°C	16·4°C	13·8 mm
**Piura**	2,077,039	58·1	31·9	27·8°C	16·1°C	25·4 mm
**Tumbes**	255,712	55·0	64·6	29·7°C	19·4°C	27·4 mm

Section 3   Further model framework details

The following section provides additional details to the description of the modelling framework presented in Section 2.3.

Section 3.1   Population Offset

For each year and each department, we had access to mid-year population estimates published by the INEI (National Institute of Statistics and Information of Peru) on 30th June of each year [20].

Then, for each month ($t = 1, \dots 140$), we linearly interpolated between the mid-yearly population values to avoid improbable population fluctuations which would otherwise arise in a single month. In Equation 2.1, $\log(P_{it})$ meant the natural logarithm of the (interpolated) population of department i at time t divided by 100,000. The population offset enabled our modelling of the logarithm of the dengue incidence rate (DIR) via $\log(\eta_{it})$.

Section 3.2   Fixed effects

Our fixed effects, expressed as a matrix of two columns $\boldsymbol{x_i}$ in Equation 2.1, comprised of; i) our seasonality indicator for the summer months (= 1 for months of December to April, and 0 otherwise) common to each department and ii) the our momentum oscillator; Relative Strength Index (RSI), lagged by one month. The objective of the simple binary seasonality variable was to capture seasonal trends of the summer months which are common across the departments. Whilst our climatic variables accounted for the influence of environmental conditions on vector abundance and incidence rates, the seasonality variable provided a means of accounting for possible population transience and increased outdoors activity during summer months. Meanwhile, the proposed concept of the RSI variable was motivated by its usage as a technical indicator in financial markets to measure the strength and momentum of price movements [32]. For each department, we employed the RSI based on the smoothed moving average of the previous three month's DIRs. Precisely, the formula used for RSI was:

$$\text{RSI} = 100 - \frac{100}{(1 + \text{RS})}$$

where RS is the *relative strength*, defined as the smoothed ratio of average gains (sum of the upward DIR movements over the past three months, divided by three - the number of months) over the average losses (sum of the downward DIR movements over the past three months, divided by three). Here, by using the RSI over

the past three months, we aimed to track the recent momentum in DIR movements and provide intuition for future possible DIR movements.

Section 3.3 Temporal random effects

Our temporal random effects comprised of: i) a department-specific, cyclic Random Walk (RW) model of order one for the monthly random effects, and ii) department-specific yearly, exchangeable random effects. First, our cyclic RW(1) prior distribution allowed for each department's current month's DIR to depend upon the observed DIR of the previous month, thus introducing temporal autocorrelation into our model structure. The cyclic aspect allowed for the DIR in January of a given year to be related to the DIR observed in the preceding month (December) in the previous year. We also experimented with the usage of a RW(2) prior distribution (a random walk of order two) which would allow for the current DIR observation to depend upon the two previously observed states (two previous month's DIR). Second, our department-specific yearly, exchangeable random effects meant that we adjusted for the possibility that each department may have observed distinct year-to-year heterogeneities such as changes to vector control programmes or varying reporting rates (possibly due to overwhelming of health services or alternatively improvements in epidemiological surveillance). Yearly random effects common across departments were found to be too coarse to enable sufficient description of the year-to-year variability within departments.

Section 3.4 Spatiotemporal random effects

Our decision to employ the BYM2 model, an adapted Bayesian version of the Besag-York-Mollié (BYM) model, was motivated by two reasons; i) to adequately capture spatial autocorrelation between the three neighbouring departments, and ii) to account for any non-spatial heterogeneity [33]. In particular, we used a version of the BYM model which employed Penalised Complexity prior distributions (to guard against overfitting) and divided the model into the two components; a spatially structured component and a spatially unstructured component [34]. Spatial autocorrelation means that even after adjusting for several covariates (such as climatic influences), we expected that the case counts (and DIRs) in neighbouring departments would not be wholly independent and would share similarities. The inclusion of non-spatial heterogeneity was useful in terms of adjusting for several of the unobserved confounders (discussed in Section 4).

In general, the BYM2 model comprises of two hyperparameters; a marginal precision parameter τ and a mixing parameter ϕ. Specifically, our penalised complexity prior on the precision parameter τ meant that $P(\frac{1}{\tau} > 0 \cdot 5) = 0 \cdot 01$. In terms of our model formula (Equation 2.1), the mixing parameter ϕ controlled the proportion of variation explained by our spatially structured component ($\mathbf{u}$) and our spatially unstructured component ($\mathbf{v}$). The spatially structured component ($\mathbf{u}$) was modelled as a conditional autoregressive process, and represented spatial variation not accounted for by our various other model components. The spatially unstructured component ($\mathbf{v}$) was modelled using an independent and identically distributed Gaussian process.

Section 3.5 Specification of climate-based DLNMs

Our model framework employed distributed lag non-linear models (DLNMs) to capture the potentially non-linear and delayed effects of climatic variables on dengue incidence rates. DLNMs are a flexible modelling framework which are based on the statistical concept of a *crossbasis*; a bi-dimensional functional space comprised of a combination of two sets of basis functions which represent the relationships with the response in the dimensions of the predictor and lags respectively [35]. In particular, usage of the DLNM framework involves the simultaneous specification of i) non-linearity in the exposure-response relationship and ii) delayed effects in the lag-response dimension. The specification is achieved via the *cross-basis*, thus creating an exposure-lag-response function.

For our model framework, using the R package `dlnms`, we employed basis splines (B-splines) for our two sets of basis functions, which were combined to generate our cross-basis functions [35]. Specifically, in the exposure-response dimension, we specified B-splines with two equally-spaced knots to enable sufficient flexibility in the modelling of the potentially complex relationship between the climatic exposure and response. For each climatic variable, the set of basis functions in the exposure-response were centred on the mean observed value of the climatic variable. In the lag-response dimension (up to four months), we allowed for complexity in the delayed effects of climatic variables by specifying a single knot at two months. Sensitivity analyses considered various alternative specifications such as the specification of a single knot a one or three months in lag-response dimension, a simpler specification of a single knot in the exposure-response dimension, and the usage of natural splines (instead of B-splines). We assessed model performance with varying inclusions of the DLNMs for the six climatic variables (which were described in Section 2.1 and visualised in Fig G).

Section 4 Figures

Section 4.1 Exploratory analyses

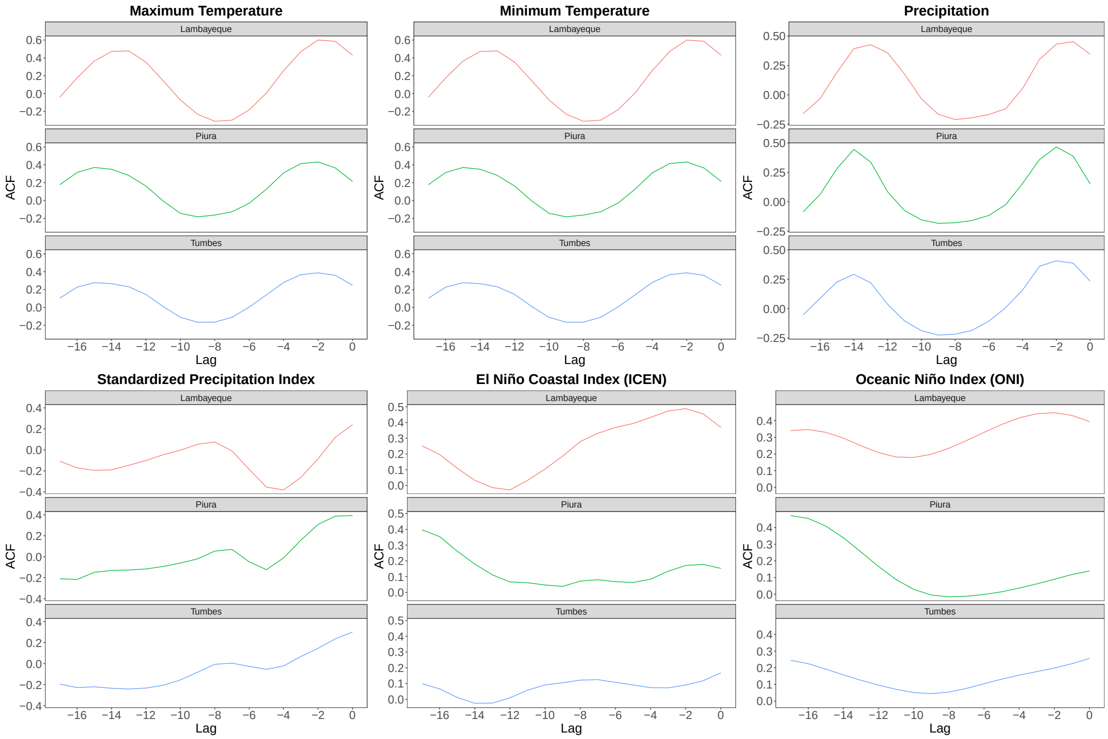

Figure A: **Exploratory analysis of climate-dengue relationships.** Cross-correlation plots for monthly lags of six climatic variables and the monthly dengue incidence rate (DIR) per 100,000 in each of the three studied departments. The plots depict a strong degree of shared similarity across departments in the timing and nature of relationship between the climate exposure and DIR. Negative lags correspond to lead times in months.

Section 4.2   Sensitivity analyses

Section 4.2.1   Climatic inference without seasonality and momentum effects

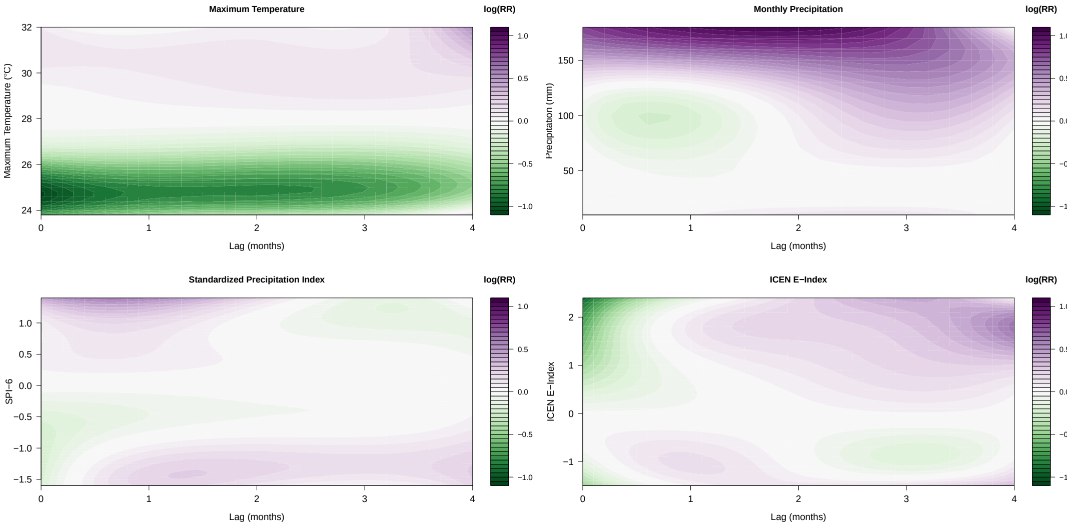

Figure B: **Omitting RSI and seasonality effects, exposure-lag-response relationships between climatic variables and dengue incidence.** Plots of relative risk (RR), on a logarithm scale, for the included DLNMs (Distributed Lag Non-linear Models) in a climate-based Bayesian hierarchical model for dengue incidence fitted to the entire period of 140 months, where RR is defined relative to the risk induced by the mean observed value of each climate variable. Log RR values greater than 0 (pink to purple) correspond to heightened relative risk of dengue incidence, whilst values less than 0 (green) correspond to reduced RR. The four climatic variables were included in the model via DLNM specifications alongside spatial random effects, temporal random effects, and spatiotemporal random effects, but *not* including our usual fixed effects (of momentum and seasonality).

Section 4.2.2 Climatic inference without maximum temperature effects

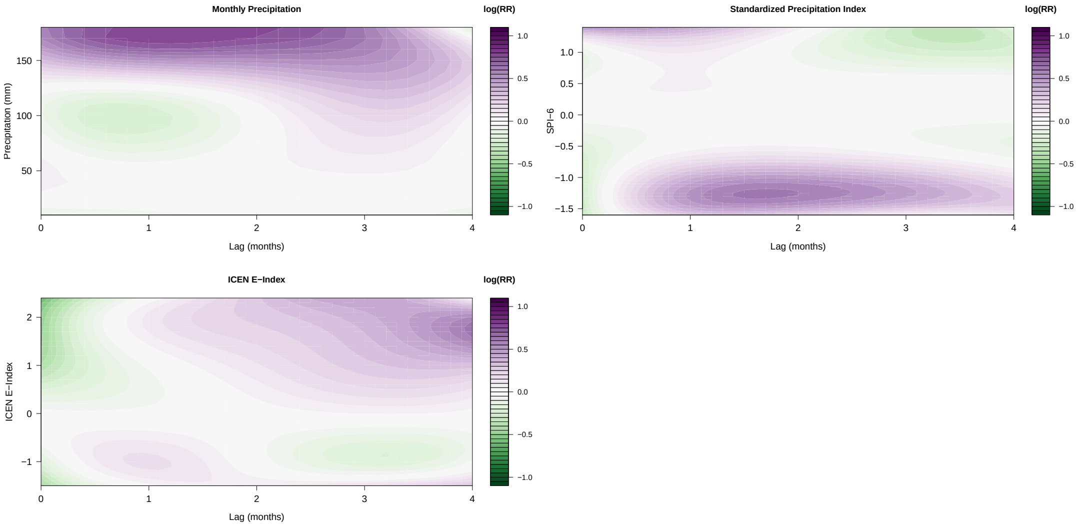

Figure C: **Omitting maximum temperature effects, exposure-lag-response relationships between climatic variables and dengue incidence.** Plots of relative risk (RR), on a logarithm scale, for the included DLNMs (Distributed Lag Non-linear Models) in a climate-based Bayesian hierarchical model for dengue incidence fitted to the entire period of 140 months, where RR is defined relative to the risk induced by the mean observed value of each climate variable. Log RR values greater than 0 (pink to purple) correspond to heightened relative risk of dengue incidence, whilst values less than 0 (green) correspond to reduced RR. The three climatic variables were included in the model via DLNM specifications alongside spatial random effects, temporal random effects, and spatiotemporal random effects, and fixed effects (of momentum and seasonality).

Section 4.2.3 Climatic inference without precipitation effects

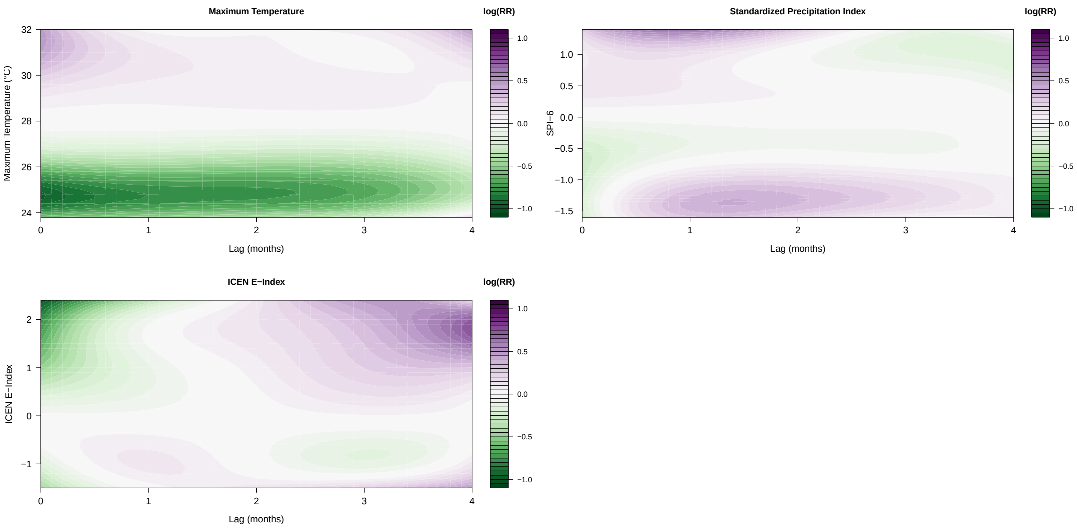

Figure D: **Omitting precipitation effects, exposure-lag-response relationships between climatic variables and dengue incidence.** Plots of relative risk (RR), on a logarithm scale, for the included DLNMs (Distributed Lag Non-linear Models) in a climate-based Bayesian hierarchical model for dengue incidence fitted to the entire period of 140 months, where RR is defined relative to the risk induced by the mean observed value of each climate variable. Log RR values greater than 0 (pink to purple) correspond to heightened relative risk of dengue incidence, whilst values less than 0 (green) correspond to reduced RR. The three climatic variables were included in the model via DLNM specifications alongside spatial random effects, temporal random effects, and spatiotemporal random effects, and fixed effects (of momentum and seasonality).

Section 4.2.4 Climatic inference without drought indicator effects

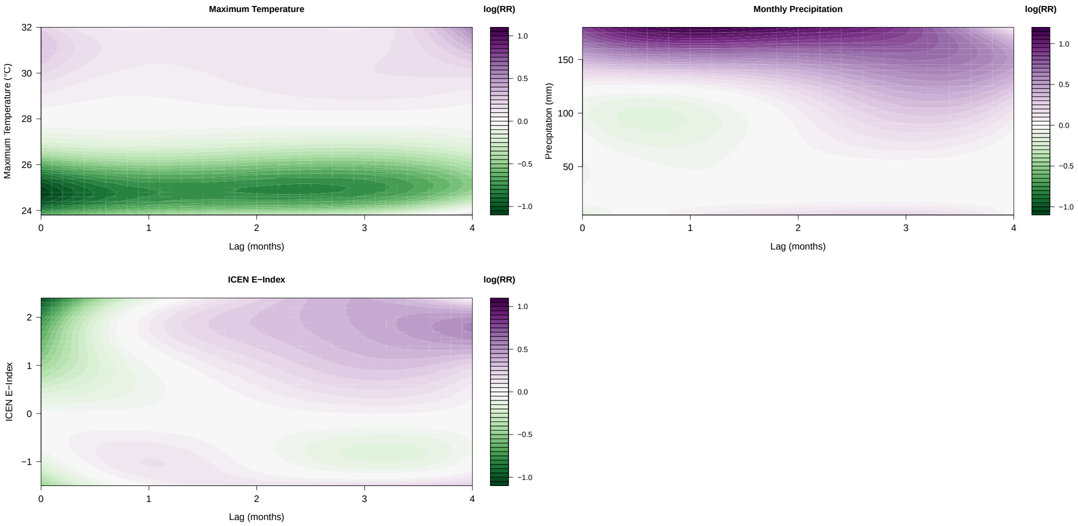

Figure E: **Omitting the drought indicator, exposure-lag-response relationships between climatic variables and dengue incidence.** Plots of relative risk (RR), on a logarithm scale, for the included DLNMs (Distributed Lag Non-linear Models) in a climate-based Bayesian hierarchical model for dengue incidence fitted to the entire period of 140 months, where RR is defined relative to the risk induced by the mean observed value of each climate variable. Log RR values greater than 0 (pink to purple) correspond to heightened relative risk of dengue incidence, whilst values less than 0 (green) correspond to reduced RR. The three climatic variables were included in the model via DLNM specifications alongside spatial random effects, temporal random effects, and spatiotemporal random effects, and fixed effects (of momentum and seasonality).

Section 4.2.5   Climatic inference without El Niño effects

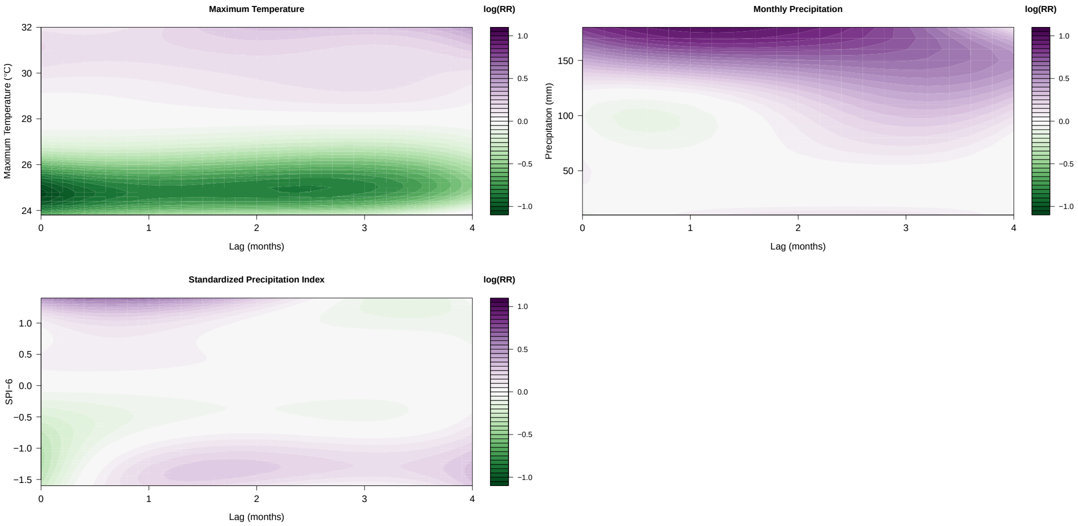

Figure F: **Omitting El Niño effects, exposure-lag-response relationships between climatic variables and dengue incidence.** Plots of relative risk (RR), on a logarithm scale, for the included DLNMs (Distributed Lag Non-linear Models) in a climate-based Bayesian hierarchical model for dengue incidence fitted to the entire period of 140 months, where RR is defined relative to the risk induced by the mean observed value of each climate variable. Log RR values greater than 0 (pink to purple) correspond to heightened relative risk of dengue incidence, whilst values less than 0 (green) correspond to reduced RR. The three climatic variables were included in the model via DLNM specifications alongside spatial random effects, temporal random effects, and spatiotemporal random effects, and fixed effects (of momentum and seasonality).

Section 4.3 Spatiotemporal climate trends

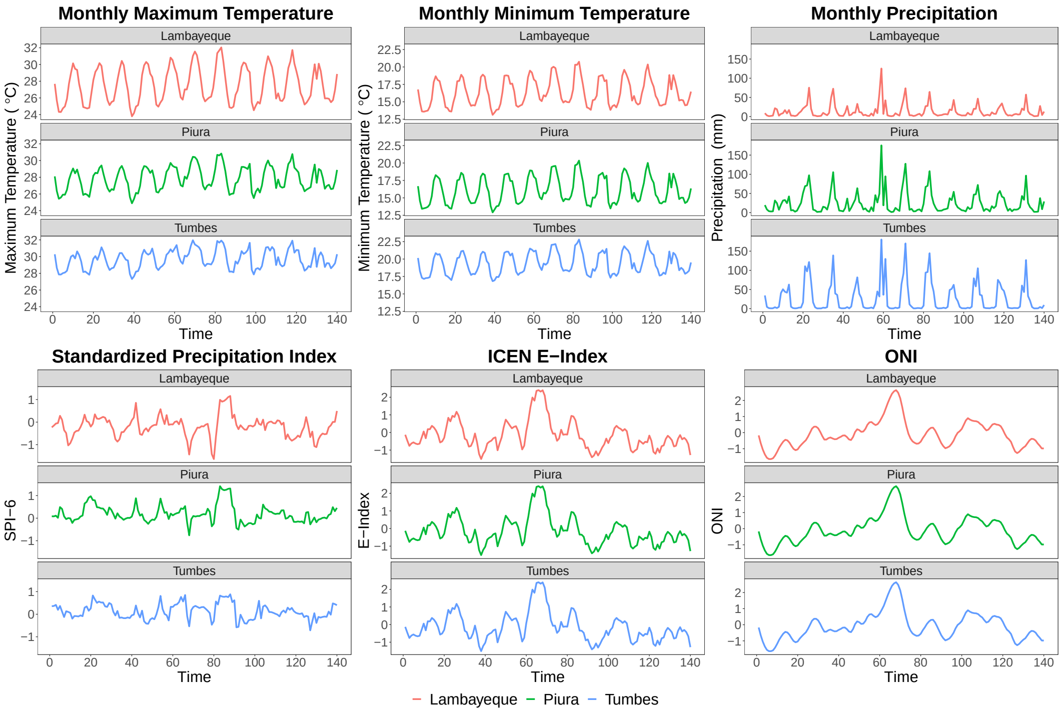

Figure G: **Historical climatic time series.** Exploratory plots which depict the monthly climatic data in Piura, Tumbes and Lambayeque over 140 months from May 2010 to December 2021. Both indicators for El Niño events (ICEN and ONI) are common across the three departments.

Section 4.4 Seasonality in dengue incidence rates

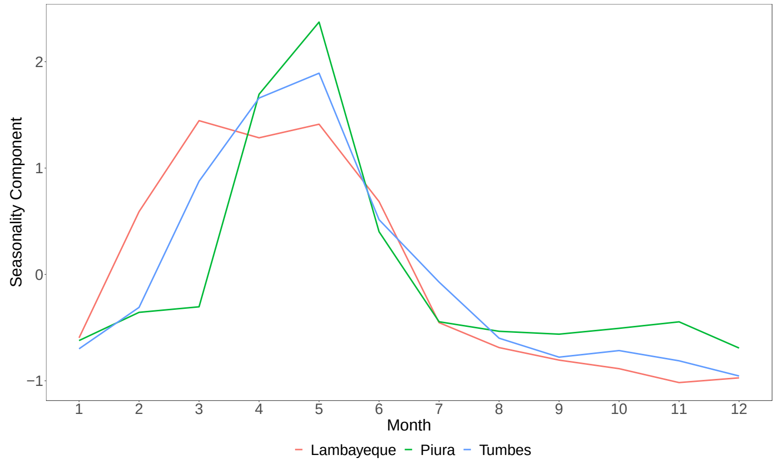

Figure H: **Department-specific seasonality.** A decomposition of the seasonality component in dengue incidence rates in each of the departments, calculated using the `bfast` package in R, which iteratively decomposes a time series into trend and seasonality components whilst detecting abrupt changes within the components. [55, 56] The results of the `bfast` algorithm indicate similarity in the seasonal trends in dengue incidence rates across each of the three departments.

Section 4.5 Cumulative risk induced by climatic variables

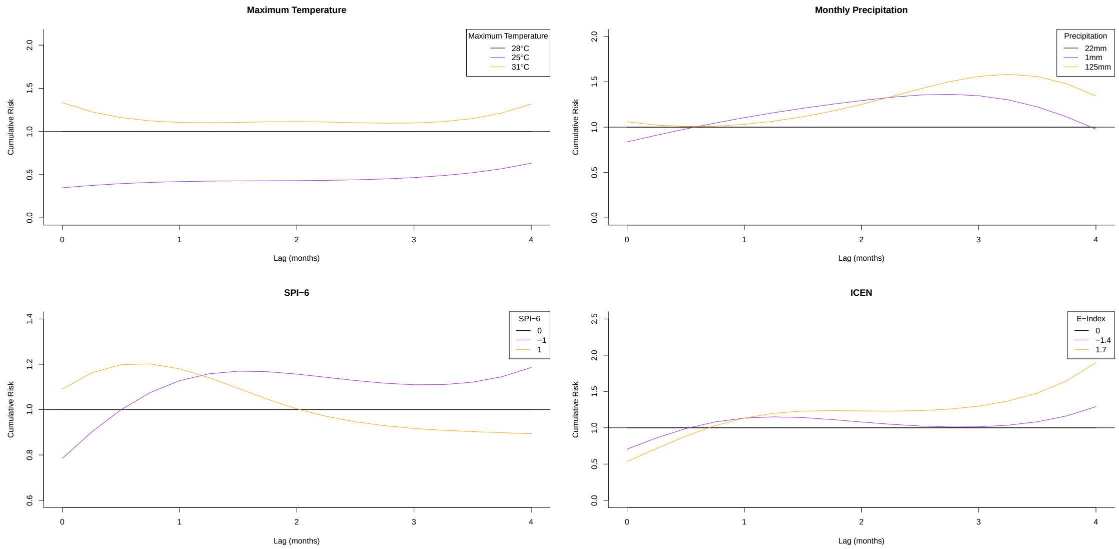

Figure I: **Cumulative association between climatic variables and DIR.** Plots of cumulative risk for the included DLNMs in climate-based Bayesian hierarchical model for dengue incidence, where cumulative risk is defined relative to the cumulative risk induced by the mean observed value of each climate variable. Cumulative risk values greater than 1 correspond to heightened cumulative risk of dengue incidence, whilst values less than 1 correspond to reduced cumulative risk. For example, as cumulative risk was negative for a monthly precipitation of 1mm at the current lag, then exposure over one month to dry conditions was associated with reduced risk, whilst positive values of cumulative risk at two months indicates that the exposure over two months resulted in a increased cumulative risk in dengue incidence. Note that in each sub-plot, we define a high and low value of the climatic variable, and the cumulative risk at each lag is defined relative to the cumulative risk induced by the mean value of the climatic variable.

Section 4.6 Retrospective modelling in-sample predictive fit

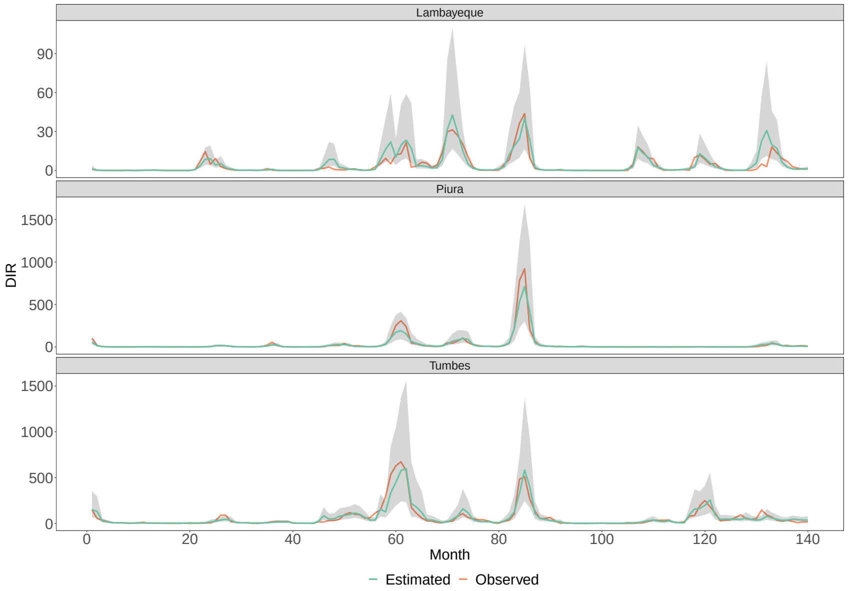

Figure J: **In-sample predictive performance of our Bayesian spatiotemporal model**. The DIR (Dengue Incidence Rate) time series (gold) for each department is plotted alongside the posterior median estimate (forest green) for each observation and the corresponding estimated 95% credible intervals (shaded grey). Fig 4 is the corresponding figure for the cross-validation setting which display the estimated out-of-sample predictive performance and thus, approximates the model's generalisability.

Section 4.7 Estimated yearly random effects by department

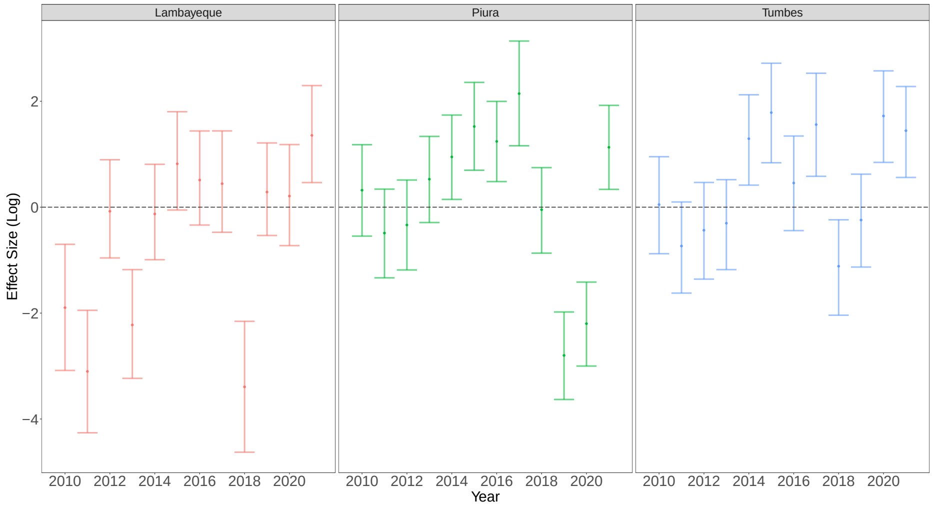

Figure K: **Estimated posterior distributions of department-specific yearly random effects**. The visualisation displays the posterior median estimate (circles) of the effect size (on a logarithmic scale) of the yearly random effect for each department, whilst the errorbars capture the 95% credible intervals. Note that the effect size can be interpreted as the contribution of the department-specific yearly random effect to the logarithm of DIR (Dengue Incidence Rate) observations within each year. The variability across departments in the yearly random effects is indicative of the differences in trends of year-to-year heterogeneities which prevented a shared yearly random effect (for each department) providing sufficient explanatory power.

Section 4.8 Estimated monthly random effects by department

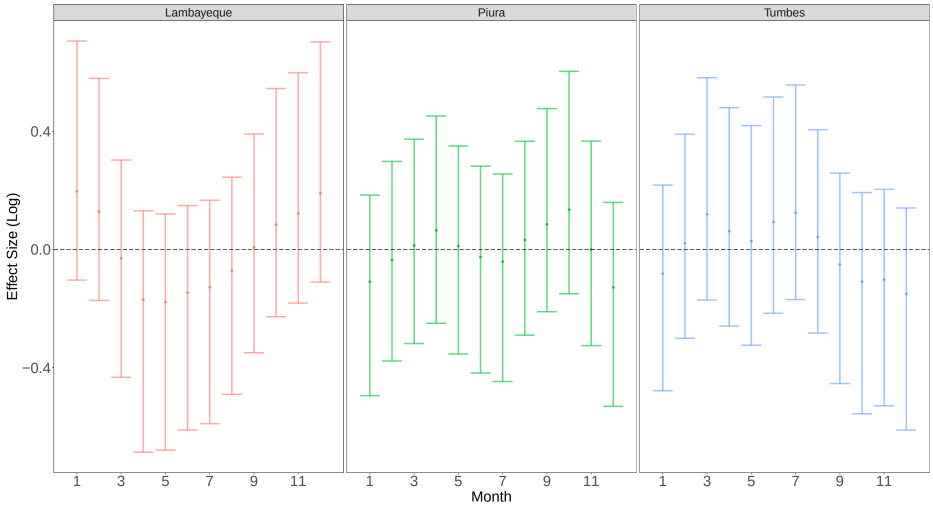

Figure L: **Estimated posterior distributions of department-specific monthly random effects**. The visualisation displays the posterior median estimate (circles) of the effect size (on a logarithmic scale) of the monthly random effect for each department, whilst the errorbars capture the 95% credible intervals. Note that the effect size can be interpreted as the contribution of the department-specific monthly random effect to the logarithm of DIR (Dengue Incidence Rate) observations across the years. Note that the contribution to DIR is in addition to the common seasonality indicator variable for summer months.

Section 4.9 Estimated spatially structured random effects by department

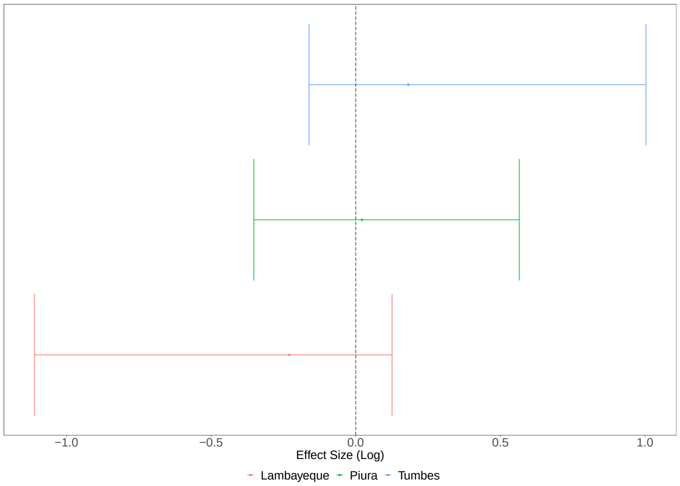

Figure M: **Estimated posterior distributions of department-specific structured spatial component effects**. The visualisation displays the posterior median estimate (circles) of the effect size (on a logarithmic scale) of the spatially structured random effect component of the BYM2 model for each department, whilst the errorbars capture the corresponding 95% credible intervals. Note that the effect size can be interpreted as the contribution of the department-specific spatial random effect to the logarithm of DIR (Dengue Incidence Rate) observations across the years.

Section 4.10 Estimated effect of momentum indicator

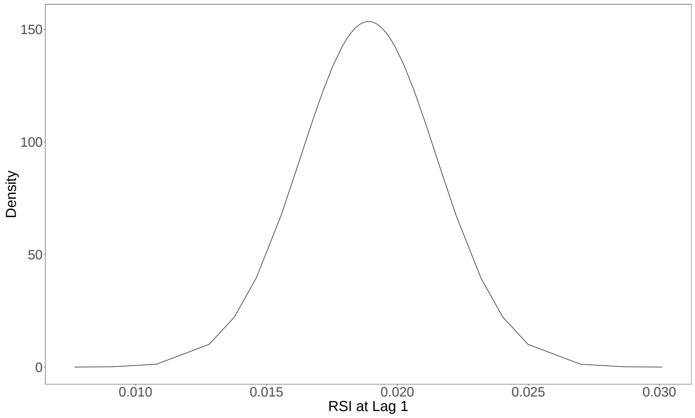

Figure N: **Estimated posterior distributions effects of momentum indicator**. The visualisation displays the posterior distribution of the effect size (on a logarithmic scale) of the lagged momentum indicator. Note that the effect size can be interpreted as the contribution of the lagged momentum indicator, calculated for three months of DIR (Dengue Incidence Rate) observations, to the current logarithm of DIR. We use the lagged momentum indicator as this ensures that the covariate does not include the response (DIR) itself.

Section 4.11 Visualisation of climate-based out-of-sample errors

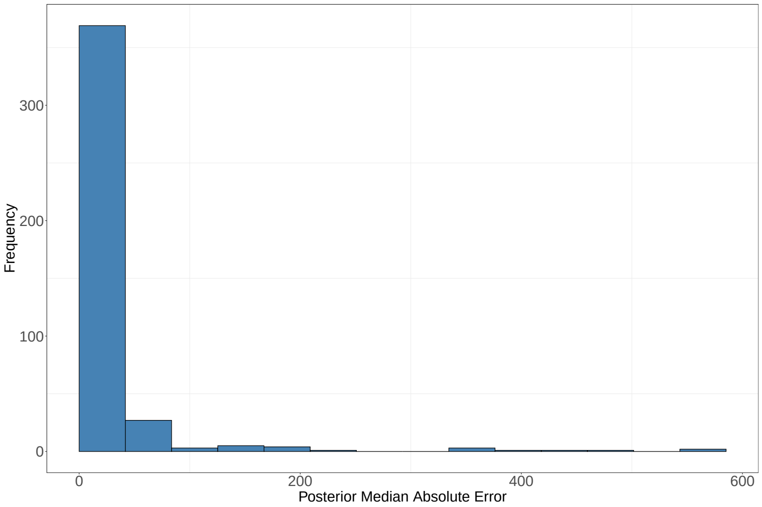

Figure O: **Leave-one-out cross-validation errors visualisation**. The histogram depicts the posterior median estimate of the absolute error for each observation, which was derived individually for observations by computing the median absolute difference between the posterior predictive value and the corresponding observed DIR (Dengue Incidence Rate). 89.6% of the posterior median estimates (n = 180) for the absolute forecast errors are less than 50 per 100,000.

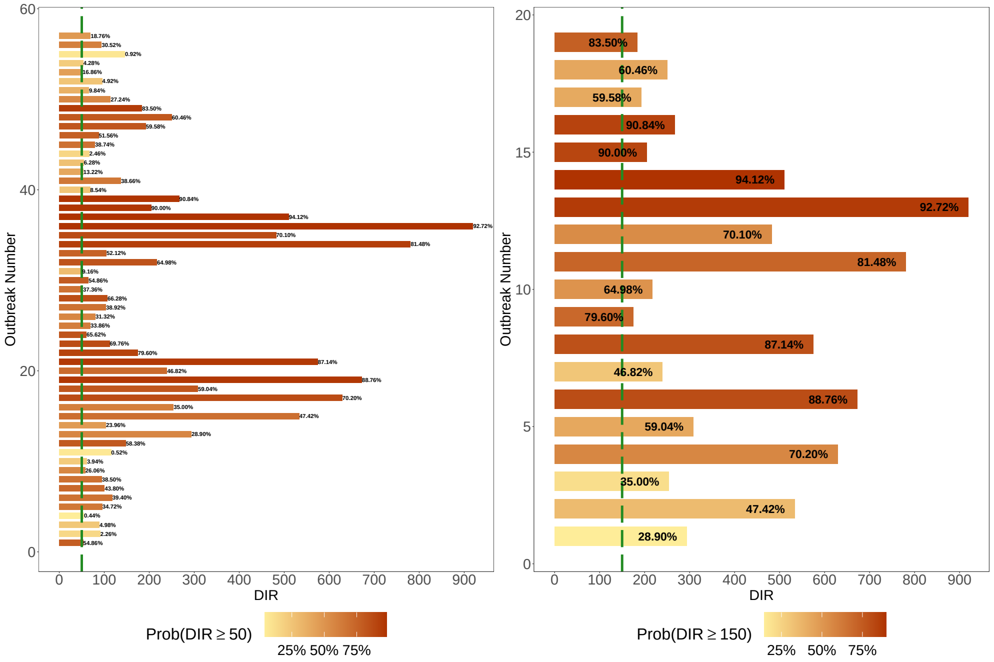

Figure P: **Retrospective modelling outbreak detection.** Among the observations with DIR (Dengue Incidence Rate) greater than 50 (left) and greater than 150 (right) per 100,000, the plots depict posterior probabilities of DIR exceeding thresholds (green) of 50 and 150 respectively, obtained via a leave-one-time-point-out fitting of models. The plots capture the model framework's sensitivity in estimating substantial out-of-sample dengue outbreaks. Within the two subsets of observations, the median posterior probabilities of exceeding 50 per 100,000 and 150 per 100,000 are 0·68 and 0·57 respectively. To also display the false positive rate and thus ensure a representative picture of our model's outbreak detection capabilities, Fig Q is the analogous visualisation for the corresponding posterior probabilities of the observations with DIR less than the thresholds of 50 and 150 per 100,000.

Table C: **Summary statistics for LOOCV outbreak detection performance.** Out-of-sample observations (n = 417), across 2010 to 2021, were classified as being predicted true outbreaks such that DIR exceeded 50 (or 150) per 100,000 if the posterior probability of DIR exceeding 50 (or 150) per 100,000 was greater than a calibrated cut-off probability of 0.16 (or 0.13). *True Positive* measures the hit rate, or equivalently the proportion of true outbreaks correctly detected, whilst *False Positive* measures the proportion of non-outbreaks incorrectly classified as outbreaks. *Accuracy* represents the proportion of observations whose outbreak classification matched the true observed state (such as a predicted outbreak coinciding with an outbreak). *Precision* measures the proportion of predicted outbreaks which were true outbreaks. Finally, the *AUC* is the area under the Receiver Operating Characteristic (ROC) curve, is used as a measure of our model's skill for distinguishing between outbreaks and non-outbreaks, and here, represents an upper bound on the model's retrospective performance (due to calibration of our cut-off probability for outbreak classification). 95% confidence intervals (CIs) for summary statistics were derived using exact binomial confidence limits [57, 58], with the exception of the 95% CI for AUC being calculated using 2,000 bootstrap replicates.

**Outbreak Threshold**	True Positive	False Positive	Accuracy	Precision	AUC
**DIR ≥ 50**	0·93	0·13	0·88	0·54	0·90
95% CI	(0·83, 0·98)	(0·09, 0·16)	(0·85, 0·91)	(0·44, 0·64)	(0·87, 0·94)
**DIR ≥ 150**	1·00	0·06	0·94	0·43	0·97
95% CI	(0·82, 1·00)	(0·04, 0·09)	(0·91, 0·96)	(0·28, 0·59)	(0·96, 0·98)

Section 4.12 Reliability of retrospective modelling outbreak detection

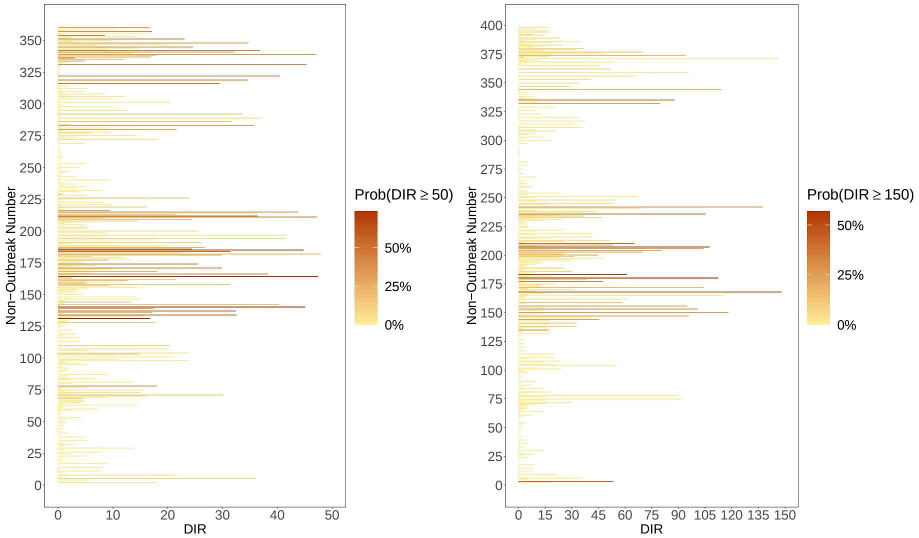

Figure Q: **Reliability of estimated out-of-sample outbreaks.** Among the observations with DIR (Dengue Incidence Rate) less than 50 (left) and DIR less than 100 (right), the plots depict posterior probabilities of forecasted DIR exceeding thresholds of 50 per 100,000 (left) and 150 per 100,000 (right). The plots visualise the model's false positive rate in terms of forecasting severe dengue outbreaks. In the subset of observations with DIR less than 50, the mean posterior probability of exceeding 50 is 0·05, whilst the maximum posterior probability of exceeding 50 is 0·60. Similarly, in the subset of observations with DIR less than 150, the mean posterior probability of exceeding 50 is 0·08, whilst the maximum posterior probability of exceeding 50 is 0·43. In terms of the true positive rate (or hit rate), Fig P is the analogous visualisation for the corresponding posterior probabilities of the observations with DIR greater than the thresholds of 50 and 150.

Section 4.13 Visualisation of climate-based forecasting errors

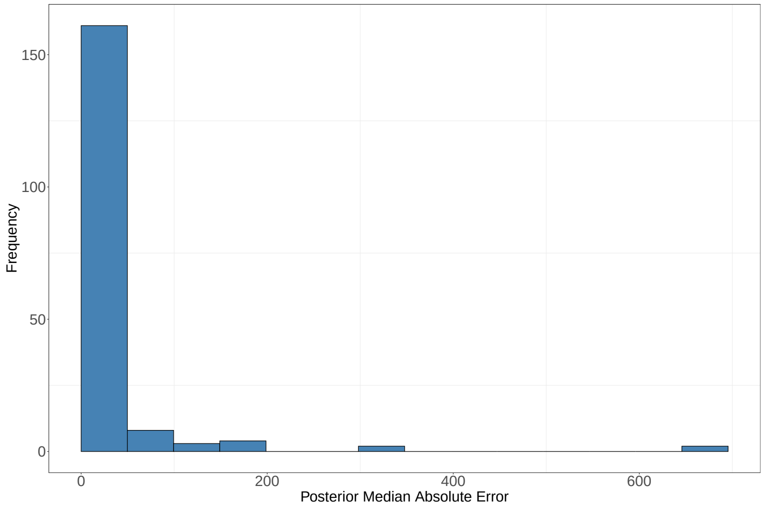

Figure R: **Forecasting errors visualisation**. The histogram depicts the posterior median estimate of the absolute error for each observation, which was derived individually for observations by computing the median absolute difference between the posterior predictive value and the corresponding observed DIR. 89.2% of the posterior median estimates (n = 180) for the absolute forecast errors are less than 50 per 100,000.

Section 4.14 Reliability of forecasting outbreak detection

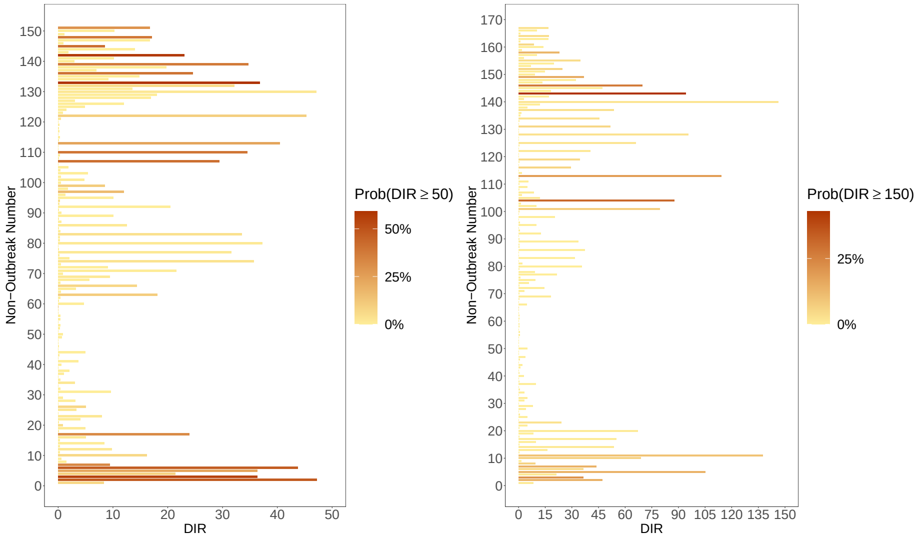

Figure S: **Reliability of forecasted outbreak detections.** Among the observations with DIR less than 50 (left) and DIR less than 100 (right), the plots depict posterior probabilities of forecasted DIR exceeding thresholds of 50 per 100,000 (left) and 150 per 100,000 (right). The plots visualise the model's false positive rate in terms of forecasting severe dengue outbreaks. In the subset of observations with DIR less than 50, the mean posterior probability of exceeding 50 is 0·05, whilst the maximum posterior probability of exceeding 50 is 0·59. Similarly, in the subset of observations with DIR less than 150, the mean posterior probability of exceeding 150 is 0·02, whilst the maximum posterior probability of exceeding 50 is 0·43. In terms of the true positive rate (or hit rate), Fig 6 is the analogous visualisation for the corresponding posterior probabilities of the observations with DIR greater than the thresholds of 50 and 150.

Section 4.15 Non-model-based simple forecasting outbreak detection

Table D: **Summary statistics for non-model-based forecasting outbreak detection across 2018 to 2021.** DIR (Dengue Incidence Rate) observations (n = 180) were classified one month ahead of time as being predicted true outbreaks with DIR exceeding 50 (or 150) per 100,000 *only* if the previous month's DIR had exceeded 50 (or 150) per 100,000. The performance metrics below, and associated 95% confidence intervals (CIs), each have the same interpretation as defined in Table C. Classification of outbreaks below can be compared to our model-based classification of future outbreaks in Table 1, where 100% of all outbreaks with DIR ≥ 50 (or 150) per 100,000 were correctly forecasted one month ahead of time, alongside higher overall forecasting skill implied by larger Area Under the Curve (AUC) values.

**Outbreak Threshold**	True Positive	False Positive	Accuracy	Precision	AUC
**DIR ≥ 50**	0·80	0·03	0·94	0·80	0·89
(95% CI)	(0·61, 0·93)	(0·01, 0·07)	(0·90, 0·97)	(0·61, 0·93)	(0·81, 0·97)
**DIR ≥ 150**	0·70	0·02	0·97	0·70	0·84
95% CI	(0·35, 0·93)	(0·00, 0·05)	(0·91, 0·98)	(0·35, 0·93)	(0·69, 0·99)
