# Supplementary material for: Climate-based modelling and forecasting of dengue in three endemic departments of Peru
Source: PLoS Negl Trop Dis. 2024 Dec 4;18(12):e0012596. doi: 10.1371/journal.pntd.0012596 (PMC11649132; doi:10.1371/journal.pntd.0012596)
Supplement: Attachment — Submitted filename: dengue_peru_reviewers_response.pdf [file pntd.0012596.s003.pdf]

Cathal Mills,
Department of Statistics,
University of Oxford,
Oxford,
OX1 3LB

12th July 2024

Dear reviewers,

We thank you for your detailed feedback which helped us to enhance the overall quality of our analysis and the clarity of our work.

Briefly, in response to the primary concerns of reviewers (which focused on additional setting-specific details), we have now added throughout more details (across the introduction, methodology, results, and discussion), generated new accompanying visualisations to illustrate our findings and conclusions, and contextualised our results and discussion both in time and space.

We believe that the present revision including several additional analyses along with many clarifications is substantially improved and will provide readers with a clear, interpretable analysis of the climatic influences on dengue transmission in a region with substantial recent dengue burden.

Please find below a point-by-point response to your comments.

1. **Comment 1:** "The first is a more detailed description and visualization of the dengue data. The Dengue Incidence rates are shown for model comparison in Fig. 4, for example, but the data are really hard to see. Perhaps the Incidence rates and/or even the crude incidences could be given, just so that we can better understand the actual burden of dengue."
   Response: As suggested, we now provide more contextual information about the dengue surveillance data. In particular, we have now added a new visualisation to show the burden of dengue over time and across the individual departments. These modifications help put our results into context and provide the reader with an initial picture of the epidemic situation over time.

2. **Comment 2:** "The second is the public health definition of a dengue case. Are these just confirmed cases by laboratory methods, and/or probable cases as well and/or even suspected cases? It would be useful to know what methods the local public health authorities use.
   Response: We now provide more thorough descriptions of the public health definitions of dengue cases. This includes the criteria for both probable and confirmed cases, and our treatment of each of these types of cases.

3. **Comment 3:** "The third is some more detailed information on the three study sites and reason for their selection. The latter seems to be related to the desire to include the El Niño phenomenon, but it's not clear why a non-coastal site wasn't taken as well as this phenomenon can have strong effects not just on the coast. In addition, although the scale selected is quite coarse, insofar as dengue is particularly problematic in urban settings, it would be useful to know a little more of the urban/rural nature of these departments.

Giving overall population number and density alone are not very useful; 99% of the population could be living in the major city and no one in the rural settings and vice versa."
Response: As suggested, we now outline our reasoning for our selection of the study sites in the context of our research objectives, describe the percentages of populations living in urban areas, and put our discussion pieces into greater setting-specific context.

4. **Comment 4:** "In addition, mosquito oviposition sites through water storage etc are mentioned in general terms in the Introduction and Discussion, but it would be useful to know specifically for these departments (in the Discussion?).

   Response: We have now added further details of water storage practices in both the introduction and discussion, with a specific focus of the impacts of these practices in the studied departments.

5. **Comment 5:** "Finally, as the paper is about forecasting, albeit at one month lead time, some mention of what is the actual vector control strategy and whether such interventions are taken at the national, departmental or municipal levels.
   Response: As suggested, we have now incorporated details of the vector control strategies undertaken in the departments, and which authorities implement these strategies.

6. **Comment 6:** "Italics for genus and species names throughout."
   Response: We have now included italics for genus and species throughout the manuscript (including the supplementary material).

7. **Comment 7:** **"**Solid waste (especially plastics) provide a huge number of oviposition sites during the rainy months whereas water storage are predominant sites during the dry season. Ae. aegypti has been found to oviposit in sewage systems but this is relatively minor in comparison to solid waste, especially as this species prefers not overly organic water sites; it's not like many Culex species that do prefer sewage related sites."
   Response: We appreciate this useful insight which has now been incorporated into our discussions of the effects of precipitation and drought.

8. **Comment 8:** **"**Meteorological variables. A pretty minor point, but diurnal temperature range (DTR) has been consistently shown to have a major effect on dengue incidence. However, given the excellent fit from the other temperatures variables, it's hard to see how this can be improved and would unlikely help extend the lag time beyond one month as DTR tends to impact on current or one month lag dengue incidences. Just a thought if the authors fancy a look"
   Response: We agree that diurnal temperature range is another useful way to estimate the effects of temperature variability on dengue incidence. In the current framework, we have included only one temperature variable (for causal reasons) which reflects temperature extrema. We have now briefly discussed that investigating such temperature variability would be an interesting future avenue of research.

9. **Comment 9:** **"**The novel Relative strength index and use as a fixed effect. This is interesting and an alternative to the use of the previous month's dengue incidence rate that "describes" the source of infection for the current month. I'm trying to understand this biologically as

the shape of the momentum might actually indirectly mirror the extent of herd immunity in the population. Could the authors expand on this part a little, from a biological perspective?
Response: We thank the reviewers for their positive thoughts on our newly proposed momentum indicator. We have now provided further details in the manuscript about the epidemiological intuition behind this variable (i.e. approximating the epidemic curve and thus, potentially capturing one or more epidemic influences).

10. **Comment 10:** "Also the RSI, as a fixed effect, is not mentioned in the Results. Was it useful index?"
Response: We have now included the posterior distribution for the effect size of the RSI momentum indicator.

11. **Comment 11:** "Eqn. 2.1 Maybe put loge for population offset. I realize this is stated in the supplementary materials, but just to make it clear in the main text."
Response: We have now provided an additional clarification that we have used the natural logarithm for population offset. This description is included in our introduction of the modelling framework and in the captions of figures.

Finally, we did not denote the natural logarithm with $\log_e$, but only to ensure consistency with previous similar modelling frameworks (e.g. 10.1371/journal.pmed.1002613)

12. **Comment 12:** "Is the choice of cut-offs in incidence rate of 50 and 150 per 100,000 purely arbitrary or based on some background non-epidemic threshold?
Response: We have now provided a description that the outbreak thresholds were pre-specified by us, and chosen simply to indicate differing magnitudes of outbreaks.

13. **Comment 13:** "I'm a bit concerned on the magnitude of the 95% credible intervals in Fig. 4. I'm not familiar with the Leave-one-time-point-out cross-validation predictive performance approach and so can't say whether this is normal; it's just they look like huge over-estimates."
Response: The magnitude of the 95% credible intervals is, as noted and illustrated, rather extreme on occasion but note that these expansions only occur for settings where the monthly dengue incidence rate (DIR) is extreme for that department. In short, the seemingly excessive conservativeness occurs in months with unprecedented DIRs.

14. **Comment 14:** "First paragraph: "with existing research", but no references of such existing research are given."
Response: The existing research has now been explicitly cited in this section, to complement the subsequent references.

15. **Comment 15:** "Paragraph 2. "which is aligned with many clinical and observational studies", but no references given."

Response: We have now added the necessary citations as suggested.

16. **Comment 16:** "temperatures to influence both larvae development (as more favorable conditions must be created for enzymatic reactions and metabolic processes)". I don't think you need to specify the enzyme temperature dependent kinetics etc part in parentheses."

Response: As suggested, we have removed the expression in parentheses which detracted from our discussion points.

17. **Comment 17:** **“**and oviposition (with maximum temperature estimated to be most significant at a six-week time lag in Ecuador)” I don’t understand the relation between oviposition and the part in parentheses and is this reference Anna’s paper, ref 38?
    Response: We have provided an alternative sentence to make this sentence clearer: “The estimated risk contributions from greater maximum temperatures at different time lags (across the four months) may be due to the time required for one or more of the following; temperatures to influence both larvae development and oviposition (with maximum temperature effects on oviposition activity in Ecuador estimated to be most significant at a six-week time lag)”

18. **Comment 18:** **“**Lifecycle of 30 days. It’s a lot faster than that. Egg to adult in approx. 10 days and then first egg batch within 4-5 days.
    Response: We have now corrected the previous incorrect account of the lifecycle.

19. **Comment 19:** **“**“level, sanitation infrastructure, and access to healthcare38,48,49,50. example, poor access to water supply……”. Is this missing a “For”, ie For example?”

    Response: We have now edited this section such that the sentences now have a coherent structure.

20. **Comment 20:** “In general, whilst many of the important aspects of how climatic variables can impact mosquito populations are discussed with references to other studies, nothing is said specifically about the study sites themselves in terms of water storage etc”

    Response: We have now provided further details about the water storage practices in the studied departments and how these relate to our inferred climatic effects.

21. **Comment 21:** **“**Suppl. Material 1. Aedes aegypti, species with small “a”

    Response: As suggested, we have included the italics for genus and species names.

To conclude, we have now incorporated all of the insightful comments from both reviewers into a revised manuscript which we believe is of a substantially higher quality and is far clearer in its presentation. We are confident that the revised version of our paper will make valuable contributions to the knowledge base regarding the relationship between climatic factors and dengue incidence and to public health policy in future outbreaks. Thank you once again for your invaluable contributions to our research endeavour.

Yours faithfully,

Cathal Mills (on behalf of the authors)
