# Supplementary material for: Climate-based modelling and forecasting of dengue in three endemic departments of Peru
Source: PLoS Negl Trop Dis. 2024 Dec 4;18(12):e0012596. doi: 10.1371/journal.pntd.0012596 (PMC11649132; doi:10.1371/journal.pntd.0012596)
Supplement: Attachment — Submitted filename: Review Mills and Donnelly.docx [file pntd.0012596.s002.docx]

Review Mills and Donnelly

This paper addresses a very important issue of forecasting dengue epidemics to enable health system and mosquito intervention preparedness using climatic variables. The statistical approaches are state-of-the art and the results astonishingly good. The paper is well written and well referenced.

There are however several bits of information that would help to put the work in a global perspective.

1. The first is a more detailed description and visualization of the dengue data. The Dengue Incidence rates are shown for model comparison in Fig. 4, for example, but the data are really hard to see. Perhaps the Incidence rates and/or even the crude incidences could be given, just so that we can better understand the actual burden of dengue.
2. The second is the public health definition of a dengue case. Are these just confirmed cases by laboratory methods, and/or probable cases as well and/or even suspected cases? It would be useful to know what methods the local public health authorities use.
3. The third is some more detailed information on the three study sites and reason for their selection. The latter seems to be related to the desire to include the El Niño phenomenon, but it’s not clear why a non-coastal site wasn’t taken as well as this phenomenon can have strong effects not just on the coast. In addition, although the scale selected is quite coarse, insofar as dengue is particularly problematic in urban settings, it would be useful to know a little more of the urban/rural nature of these departments. Giving overall population number and density alone are not very useful; 99% of the population could be living in the major city and no one in the rural settings and vice versa.
4. In addition, mosquito oviposition sites through water storage etc are mentioned in general terms in the Introduction and Discussion, but it would be useful to know specifically for these departments (in the Discussion?).
5. Finally, as the paper is about forecasting, albeit at one month lead time, some mention of what is the actual vector control strategy and whether such interventions are taken at the national, departmental or municipal levels.

**Minor**

6. Italics for genus and species names throughout

**Introduction**

7. Solid waste (especially plastics) provide a huge number of oviposition sites during the rainy months whereas water storage are predominant sites during the dry season. *Ae. aegypti* has been found to oviposit in sewage systems but this is relatively minor in comparison to solid waste, especially as this species prefers not overly organic water sites; it’s not like many *Culex* species that do prefer sewage related sites.

**Methods**

8. Meteorological variables. A pretty minor point, but diurnal temperature range (DTR) has been consistently shown to have a major effect on dengue incidence. However, given the excellent fit from the other temperatures variables, it’s hard to see how this can be improved and would unlikely help extend the lag time beyond one month as DTR tends to impact on current or one month lag dengue incidences. Just a thought if the authors fancy a look.
9. The novel Relative strength index and use as a fixed effect. This is interesting and an alternative to the use of the previous month’s dengue incidence rate that “describes” the source of infection for the current month. I’m trying to understand this biologically as the shape of the momentum might actually indirectly mirror the extent of herd immunity in the population. Could the authors expand on this part a little, from a biological perspective?
10. Also the RSI, as a fixed effect, is not mentioned in the Results. Was it useful index?
11. Eqn. 2.1 Maybe put log_e_ for population offset. I realize this is stated in the supplementary materials, but just to make it clear in the main text.
12. Is the choice of cut-offs in incidence rate of 50 and 150 per 100,000 purely arbitrary or based on some background non-epidemic threshold?

**Results**

Results are very well written. 100% prediction and only 12% (& 4%) false positive rates is fantastic.

13. I’m a bit concerned on the magnitude of the 95% credible intervals in Fig. 4. I’m not familiar with the Leave-one-time-point-out cross-validation predictive performance approach and so can’t say whether this is normal; it’s just they look like huge over-estimates.

**Discussion**

The Discussion is a little less thorough.

14. *First paragraph*: “with existing research”, but no references of such existing research are given.
15. *Paragraph 2*. “which is aligned with many clinical and observational studies”, but no references given.
16. “temperatures to influence both larvae development (as more favorable conditions must be created for enzymatic reactions and metabolic processes)”. I don’t think you need to specify the enzyme temperature dependent kinetics etc part in parentheses.
17. “and oviposition (with maximum temperature estimated to be most significant at a six-week time lag in Ecuador)” I don’t understand the relation between oviposition and the part in parentheses and is this reference Anna’s paper, ref 38?
18. Lifecycle of 30 days. It’s a lot faster than that. Egg to adult in approx. 10 days and then first egg batch within 4-5 days.

*Limitations paragraph*

19. “level, sanitation infrastructure, and access to healthcare38,48,49,50. example, poor access to water supply……”. Is this missing a “For”, ie For example?
20. In general, whilst many of the important aspects of how climatic variables can impact mosquito populations are discussed with references to other studies, nothing is said specifically about the study sites themselves interms of water storage etc etc
21. Suppl. Material 1. *Aedes aegypti*, species with small “a” and
